# Predictors of Short‐Term and Long‐Term Latency After Preterm Premature Rupture of Membranes: A Retrospective Cohort Study

**DOI:** 10.1155/jp/1603915

**Published:** 2026-03-23

**Authors:** Shokoh Abotorabi, Maryam Rafiei, Solmaz Chamanara, Mark D. Griffiths, Zainab Alimoradi

**Affiliations:** ^1^ Department of Obstetrics and Gynecology, School of Medicine, Qazvin University of Medical Sciences, Qazvin, Iran, qums.ac.ir; ^2^ Psychology Department, Nottingham Trent University, Nottingham, UK, ntu.ac.uk; ^3^ Social Determinants of Health Research Center, Research Institute for Prevention of Non-Communicable Diseases, Qazvin University of Medical Sciences, Qazvin, Iran, qums.ac.ir

**Keywords:** latency duration, PPROM, preterm premature rupture of the membranes, retrospective cohort study

## Abstract

**Background:**

Preterm premature rupture of the membranes (PPROM) is the spontaneous rupture of fetal membranes before the 37th gestational week. Despite the importance of the duration of the latency period (time duration between occurrence of PPROM and childbirth), the knowledge regarding its predictive factors is limited and inconsistent.

**Aim:**

The present study is aimed at identifying the predictive factors of short‐term and long‐term latency among PPROM cases.

**Methods:**

A retrospective cohort study was conducted using hospital‐based data from a single academic tertiary care hospital between January 2018 and December 2022. Demographic and clinical characteristics of 200 participants admitted due to PPROM were collected. Latency duration was categorized into three categories: less than 48 h; 48 h–7 days (considered as short‐term latency); and more than 7 days (considered as long‐term latency). In order to investigate the predictors of short‐term and long‐term latency among PPROM cases, univariable (using ꭓ^2^, Exact F statistic, and analysis of variance) and multivariable models (multivariable multinomial logistic regression model) were used.

**Results:**

The only significant predictor of latency duration between 48 h and 7 days, compared with latency duration of < 48 h, was lower gestational age at admission (4% decrease with each day increasing in gestational age at admission, *p* = 0.02). The significant predictors of latency duration of > 7 days compared with latency duration of < 48 h were lower gestational age at admission (8% decrease with each day increasing in gestational age at admission, *p* < 0.001), normal glucose tolerance status (7.95 times increased chance, *p* = 0.003), cervical dilation of <2 cm vs. ≥ 2 cm dilatation at admission (3.27 times increased chance, *p* = 0.013), and pregnancy termination due to reaching 34 weeks of gestation (36.63 times increased chance, *p* < 0.001) compared with termination due to labor pain. These variables explained 42.3% of variance for latency duration.

**Conclusion:**

Obstetricians can expect longer latency period when PPROM cases are admitted at lower gestational age, having normal glucose tolerance status, and cervical dilation of <2 cm vs. ≥ 2 cm dilatation at admission.

## 1. Introduction

Preterm premature rupture of membranes (PPROM) is the spontaneous rupture of fetal membranes before the 37th gestational week [1]. PPROM is the result of an interaction between complex and multifaceted pathways. Several factors including maternal urogenital tract infections, behavioral factors (e.g., poor nutritional status, substance abuse, and cigarette smoking), obstetric complications (e.g., polyhydramnios, multiple gestation, prior cervical surgery, incompetent cervix, and antenatal trauma), environmental factors (e.g., stress and toxin exposure), and genetic predisposition have been proposed as precursors of PPROM [2].

PPROM occurs in 2%–7% of pregnancies [3–5], with a recurrence rate of 16%–32% [6]. PPROM leads to one‐third of all spontaneous preterm deliveries [3–5] and significantly increases the chance of neonatal mortality and morbidity than any other gestational complications [7]. The risk of neonatal and infant mortality and morbidity increases when PPROM occurs at a lower gestational age [6]. Therefore, despite the etiological cause of PPROM, gestational age is the determinant factor in the management of PPROM cases [8]. Between 24^0/7^ and 33^6/7^weeks, expectant management is recommended for reducing the complications of prematurity [9].

Expectant management aims to lengthen the time duration between occurrence of PPROM and childbirth (i.e., the latency period) [10]. The duration of the latency period is important because there is strong association between gestational age at birth and perinatal outcomes [11]. With a prolonged latency period, there is enough time to administer conventional treatments such as corticosteroids, antibiotics, and magnesium sulfate [10].

Despite the importance of latency period duration, knowledge regarding its predictive factors is limited and inconsistent [10]. The latency duration has been reported to be inversely associated with gestational age at membrane rupture [12–17], oligohydramnios [10, 14, 16], fetal growth restriction [10], twin pregnancy [13, 14], leukocyte count more than 12 × 10^9^/L, C‐reactive protein (CRP) concentration more than 5 mg/L at 7 days after PPROM [16], and nulliparity [10, 12, 15]. Latency duration has also been reported to be associated with BMI ≥ 23 kg/m^2^ and an amniotic fluid volume of ≥ 6 cm [17]. The findings of studies examining the association between cervical dilatation and length of latency period have been inconsistent. Some have found that short latency periods are associated with higher cervical dilation [10, 18], whereas others have found no association [16]. Inconsistency regarding the association of maternal age and latency duration has also been reported. Maternal age higher than 30 years has been found to be associated with both longer [15] and shorter latency period duration [17].

Having a clinically considerable latency duration is important for the timing of expectant management as the principal treatment strategy to reduce neonatal mortality and morbidity [19]. In PPROM cases, expectant management can improve neonatal survival by approximately 2% for each additional day of remaining in utero [20]. Despite the importance of latency duration, associated factors are still not well identified. Therefore, the present study aimed to identify predictive factors of short‐term and long‐term latency among PPROM cases.

## 2. Methods

### 2.1. Study Design and Setting

A retrospective single center cohort study was carried out between January 2018 and December 2022 using Kowsar Hospital information databases. Kowsar Hospital is a single specialized Obstetrics and Gynecology Hospital affiliated to Qazvin University of Medical Sciences, located in Qazvin, Iran. Kowsar Hospital is a referral center for Qazvin province. Qazvin province has six counties: Qazvin, Takestan, Abyek, Buin Zahra, Alborz, and Avaj. All complicated obstetric patients through the province are referred to Kowsar Hospital.

### 2.2. Participants and Sampling Procedure

The eligible participants were women with PPROM at the gestational age of 24–34 weeks hospitalized in Kowsar Hospital. Only patients with complete medical records were included. All hospital information regarding eligible PPROM‐related admissions to Kowsar Hospital of Qazvin from January 2018 to December 2022 was collected.

### 2.3. Patients′ Routine Care

Routine care was provided for all patients based on the national protocol for PPROM management, consistent with international guidelines [1, 4]. This included the administering of 2 g of intravenous ampicillin every 6 up to 72 h, followed by 500‐mg oral capsules of amoxicillin every 8 h up to 1 week, and 400‐mg oral tablets of erythromycin every 6 h for the next week. Moreover, to accelerate fetal lung maturity, two doses of 12‐mg betamethasone were injected on two consecutive days. Indications for childbirth include reaching 34 weeks, occurrence of spontaneous labor, fetal distress, or emergence of signs of infection. All patients were monitored daily for fetal heart rate, fever, and amniotic fluid volume. Patients′ leukocyte counts, erythrocyte sedimentation rate (ESR), and CRP were checked twice a week.

### 2.4. Variables

Latency period duration, defined as the number of days between PPROM and delivery, was categorized as less than 48 h; 48 h–7 days; and more than 7 days. This categorization aligns with previous studies and clinical relevance, where 48 h reflects the typical window for administering corticosteroids and antibiotics, and 7 days is a clinically meaningful threshold for expectant management [10, 20].

The variables of interest in the present study were age, gravid, parity, history of preterm labor (PTL), previous delivery mode, glucose tolerance status, triplet test, AF color, cervical dilatation at admission (based on vaginal examination), oligohydramnios, multiple pregnancy, CRP at admission, gestational age at admission, amniotic fluid index (AFI), white blood cell counts at admission, neutrophil counts at admission, pulse rate at admission, temperature at admission, FHR at admission, and ESR at admission. All variables were recorded based on patients′ hospital files.

CRP was dichotomized using a cutoff of > 10 mg/L, based on clinical thresholds for infection/inflammation in pregnancy commonly used in obstetric settings and supported by contemporary literature [16]. Glucose tolerance status was classified as normal or abnormal based on documented diagnosis of gestational diabetes (via 75 g OGTT at 24–28 weeks) or pregestational diabetes in medical records. The triple screening test (maternal serum alpha‐fetoprotein, hCG, and estriol) was classified as normal or abnormal based on standard hospital cutoffs; it was included as a potential marker of placental function. The reason for pregnancy termination was recorded and categorized as: reaching 34 weeks of gestation, fetal distress, chorioamnionitis, or labor pain (LP).

### 2.5. Sample Size Estimation

Given the retrospective design, all eligible cases during the study period were included (opportunistic sampling). A total of 200 PPROM cases were analyzed.

### 2.6. Ethics

The present study was approved by the Ethics Committee in Biological Research of Qazvin University of Medical Sciences (Code IR.QUMS.REC.1399.076). Written informed consent was acquired from all patients at the time of admission regarding the possibility of using their medical files′ data for future research. There was no direct contact with the patients because all the data were extracted from patients′ files. Due to the retrospective nature of the study, the need to obtain the informed consent was waived by the Ethics Committee in Biological Research of Qazvin University of Medical Sciences.

### 2.7. Statistical Analysis

Data were analyzed using SPSS 24 software. Continuous variables were summarized using means and standard deviations, and categorical variables were summarized using frequencies and percentages. Latency duration was categorized into three clinically relevant groups: < 48 h; 48 h–7 days (short‐term latency); and > 7 days (long‐term latency). Age was categorized for clinical relevance and to facilitate comparison with prior obstetric studies.

Univariable analysis was performed using chi‐square or Fisher′s exact tests for categorical variables and ANOVA for continuous variables. Normality of continuous variables was assessed using the Shapiro–Wilk test; all met ANOVA assumptions. Variables with *p* < 0.05 in univariable analysis were entered into a multivariable multinomial logistic regression model (MMLR) to identify independent predictors of latency categories, with < 48 h as the reference group.

## 3. Results

The demographic and clinical characteristics of the participants are presented in Table 1. Four‐fifths of participants were in the age group of 18–35 years (79%) and three‐fifths were multigravid (60%). History of PTL was reported by 5% of patients, and 2.5% had experienced multiple pregnancies. Univariable associations of demographic and clinical variables with latency duration are shown in Table 1. Glucose tolerance status, cervical dilatation at admission, gestational age at admission, and the reason for pregnancy termination were significant independent variables (*p* < 0.05) in the univariable analysis and entered as potential predictors in the MMLR model.

**Table 1:** Patients′ characteristics in different categories of latency duration categories and results of univariable association assessment.

Latency duration categories →		< 48 h (*n* = 46)	48 h–7 days (*n* = 60)	> 7 days (*n* = 94)	*p* value of *ꭓ* ^2^ or exact *F* statistic
Predictors ↓		*N* (%)	*N* (%)	*N* (%)	
Age group	≤ 18 years	2 (4.3)	5 (8.3)	12 (12.8)	0.56
	18–35 years	38 (82.6)	47 (78.3)	73 (77.7)	
	> 35 years	6 (13.0)	8 (13.3)	9 (9.6)	
Gravid	1	17 (37.0)	19 (31.7)	44 (46.8)	0.24
	2	17 (37.0)	18 (30.0)	23 (24.5)	
	3 ≤	12 (26.1)	23 (38.3)	27 (28.7)	
Parity	Nulliparous	24 (52.2)	30 (50.0)	50 (53.2)	0.77
	Primiparous	12 (26.1)	20 (33.3)	31 (33.0)	
	Multiparous	10 (21.7)	10 (16.7)	13 (13.8)	
History of PTL	Yes	3 (6.5)	2 (3.3)	5 (5.3)	0.77
	No	43 (93.5)	58 (96.7)	89 (94.7)	
Previous delivery mode	Nulliparous	24 (52.2)	30 (50.0)	50 (53.2)	0.78
	CS	12 (26.1)	20 (33.3)	31 (33.0)	
	NVD	10 (21.7)	10 (16.7)	13 (13.8)	
Glucose tolerance status	Normal	36 (78.3)	51 (85.0)	89 (94.7)	**0.01**
	Abnormal	10 (21.7)	9 (15.0)	5 (5.3)	
Triplet test	Normal	43 (93.5)	56 (93.3)	87 (92.6)	0.99
	Abnormal	3 (6.5)	4 (6.7)	7 (7.4)	
AF color	Clear	46 (100.0)	57 (95.0)	87 (92.6)	0.21
	Bloody	0 (0.0)	3 (5.0)	7 (7.4)	
Cervical dilatation at admission	2 cm >	20 (43.5)	32 (53.3)	73 (77.7)	**< 0.001**
	2 cm ≤	26 (56.5)	28 (46.7)	21 (22.3)	
Oligohydramnios	Yes	18 (54.5)	29 (51.8)	41 (43.6)	0.46
	No	15 (45.5)	27 (48.2)	53 (56.4)	
Multiple pregnancy	Yes	1 (2.2)	2 (3.3)	2 (2.1)	0.85
	No	45 (97.8)	58 (96.7)	92 (97.9)	
CRP at admission	> 10 mg/L	32 (71.1)	44 (75.9)	73 (77.7)	0.71
	≤ 10 mg/L	13 (28.9)	14 (24.1)	21 (22.3)	
Pregnancy termination indications	Reaching 34 weeks of gestation	2 (4.3)	7 (11.7)	38 (40.4)	**< 0.001**
	Fetal distress	12 (26.1)	9 (15.0)	13 (13.8)	
	Chorioamnionitis	4 (8.7)	9 (15.0)	7 (7.4)	
	LP	28 (60.9)	35 (58.3)	36 (38.3)	
		Mean (SD)	Mean (SD)	Mean (SD)	*p* value for ANOVA
Gestational age at admission (days)		217.54 (16.40)	211.55 (16.32)	203.83 (16.47)	**< 0.001**
Amniotic fluid index (AFI)		52.09 (38.54)	55.70 (40.46)	64.32 (42.26)	0.24
White blood cell count at admission		11,698.00 (2955.42)	11,010.34 (2091.65)	11,544.68 (2620.62)	0.33
Neutrophil count at admission (%)		78.28 (8.30)	76.90 (6.92)	76.25 (6.25)	0.28
Pulse rate at admission		90.50 (6.75)	89.64 (6.99)	89.82 (6.39)	0.79
Temperature at admission (°C)		37.05 (0.26)	37.03 (0.35)	36.97 (0.29)	0.21
FHR at admission		144.22 (9.25)	143.83 (9.40)	140.29 (15.38)	0.12
ESR at admission		34.82 (14.22)	36.02 (16.85)	32.53 (16.38)	0.41

*Note:* Gestational age at admission is presented in days. Glucose tolerance status, cervical dilatation at admission, gestational age at admission were significant variables in the univariable assessment, which were then entered in multivariable model. Bold values indicate statistical significance (*p* < 0.05).

Abbreviations: AFI, amniotic fluid index; CRP, C‐reactive protein; CS, cesarean section; ESR, erythrocyte sedimentation rate; FHR, fetal heart rate; NVD, normal vaginal delivery; PTL, preterm labor.

The results of MMLR model are shown in Table 2. For latency duration between 48 h–7 days compared with latency duration < 48 h, only gestational age at admission was a significant predictor (OR = 0.96, 95% CI: 0.93–0.99, *p* = 0.02). Pregnancy termination due to reaching 34 weeks of gestation showed a trend toward increased odds (OR = 4.02, 95% CI: 0.74–21.99, *p* = 0.11), but did not reach statistical significance in this category. For latency duration of > 7 days compared with latency duration of < 48 h, significant predictors included: gestational age at admission (OR = 0.92, 95% CI: 0.93–0.98, *p* < 0.001), normal glucose tolerance status (OR = 7.95, 95% CI: 2.01–31.44, *p* = 0.003), cervical dilation of < 2 cm versus ≥ 2 cm at admission (OR = 3.27, 95% CI: 1.29–8.29, *p* = 0.013), and pregnancy termination due to reaching 34 weeks of gestation (OR = 36.63, 95% CI: 6.86–195.50, *p* < 0.001) compared with termination due to LP. Termination due to fetal distress (OR = 0.42, 95% CI: 0.14–1.30, *p* = 0.13) and chorioamnionitis (OR = 0.51, 95% CI: 0.11–2.50, *p* = 0.41) were not statistically significant. These variables explained 42.3% of the variance in latency duration based on Nagelkerke′s pseudo R^2^.

**Table 2:** Results of multivariable multinomial logistic regression analysis.

Latency duration categories∗	Predictors		B (95% CI)	Std. error	OR	Sig.
48 h–7 days	Gestational age at admission (days)		−0.04 (0.93; 0.99)	0.02	0.96	**0.02**
	Glucose tolerance status (normal vs. abnormal)		0.77 (0.73; 6.41)	0.55	2.16	0.17
	Cervical dilatation at admission (<2 cm vs. ≥ 2)		0.27 (0.56; 3.00)	0.42	1.31	0.52
	Pregnancy termination indications	Reaching 34 weeks of gestation	1.39 (0.74; 21.99)	0.87	4.02	0.11
		Fetal distress	−0.84 (0.15; 1.25)	0.54	0.43	0.12
		Chorioamnionitis	0.23 (0.33; 4.89)	0.69	1.26	0.74
		LP	Ref			
> 7 days	Gestational age at admission (days)		−0.09 (0.93; 0.98)	0.02	0.92	**< 0.001**
	Glucose tolerance status (normal vs. abnormal)		2.07 (2.01; 31.44)	0.70	7.95	**0.003**
	Cervical dilatation at admission (<2 cm vs. ≥ 2)		1.18 (1.29; 8.29)	0.48	3.27	**0.013**
	Pregnancy termination indications	Reaching 34 weeks of gestation	3.60 (6.86; 195.50)	0.85	36.63	**< 0.001**
		Fetal distress	−0.86 (0.14; 1.30)	0.58	0.42	0.13
		Chorioamnionitis	−0.67 (0.11; 2.50)	0.81	0.51	0.41
		LP	1			

*Note:* Significant of likelihood ratio tests for effect of independent variables: Gestational age at admission (< 0.001), glucose tolerance status (0.008), cervical dilatation at admission (0.023), pregnancy termination indications (<0.001). Pseudo R^2^ based on Nagelkerke: 42.3%. Asterisk denotes the reference category: ≤ 48 h. Bold values indicate statistical significance (*p* < 0.05).

Abbreviations: CI, confidence interval; OR, odds ratio.

## 4. Discussion

The present study aimed to identify predictive factors of short‐term and long‐term latency among PPROM cases. Gestational age at admission was the only significant predictor of short‐term latency duration (48 h–7 days compared with latency duration of ≤ 48 h). The significant predictors of long‐term latency were lower gestational age at admission, normal glucose tolerance status (normoglycemic status), and cervical dilation of < 2 cm vs. ≥ 2 at admission.

In the present study, higher gestational age at admission was associated with decreased chances of short‐term and long‐term latency. This is consistent with previous evidence that reported inverse association of the latency duration with gestational age at membrane rupture [12–17, 20]. Therefore, when treating PPROM patients with a higher gestational age, shorter latency period can be expected. In this group, expectant management strategies should be started at the earliest time after admission for better therapeutic success and lower the neonatal mortality and morbidity.

Another finding in present study was the increased chance of long‐term latency when cervical dilatation at admission was less than 2 cm (vs. ≥ 2 cm). The association between cervical dilatation and length of latency period is inconsistent with findings in the extant literature. Although some studies report shortened latency periods being associated with higher cervical dilation [10, 18]—which is consistent with the present study′s findings—other studies have reported no association [16, 20]. However, it is important to note that although some studies did not observe any association between cervical dilation and latency duration [20], they did report that shorter cervical length based on transvaginal ultrasonography can be an independent predictor of delivery within 7 days among women presenting with PPROM [20, 21]. Therefore, further studies are needed to assess the clinical predictive importance of cervical dilatation of PPROM cases at admission and its association with cervical length assessed using transvaginal ultrasonography.

In the present study, reaching 34 weeks of gestation as a reason for pregnancy termination was significantly associated with longer latency (>7 days) compared with termination due to LP. This finding is clinically intuitive, as reaching 34 weeks is a planned termination criterion in PPROM management, typically occurring after successful expectant management. This variable explained additional variance in the multivariable model, highlighting the importance of distinguishing between planned and spontaneous/indicated deliveries when analyzing latency duration.

Results of present study showed that patients with normoglycemic status had a higher chance in having long‐term latency versus those with abnormal glucose tolerance status. Higher prevalence of PPROM has been reported in pregnancies complicated with diabetes [22–25], but the evidence regarding the association of glucose status and latency period has been limited and inconsistent. Although longer latency period was reported among diabetes‐free patients by Shahali et al., it was not statistically significant [26]. Kari et al. reported a significantly higher latency period among patients with gestational diabetes versus the normoglycemic group [27]. Abnormal glucose tolerance status, which is representative of diabetes in pregnancy (gestational or pregestational), is considered an important medical problem with considerable fetomaternal consequences [28]. However, it should be noted that this finding in the present study is not conclusive due to the low sample size of this subgroup, which had a wide confidence interval for the odds ratio in the multivariable model. Therefore, further studies with a larger sample size of PPROM cases with normal glycemic status and diabetes in pregnancy are needed to reach a more definitive conclusion.

### 4.1. Limitations

The present study applied a multivariable regression model to investigate the predictors of short‐term and long‐term latency duration to provide a more accurate analytic approach. However, the findings should be interpreted considering some limitations. First, the present study used a retrospective study design that might lead to not having data on some variables (e.g., cigarette smoking history, placenta position, and fetal presentation). An odds ratio index based on multivariable multinomial logistic regression analysis was used that might have overestimated the association between variables. Wide confidence intervals for odds ratios in multivariable models suggest the need for future studies with larger sample sizes in these subgroups (e.g., PPROM cases complicated with diabetes during pregnancy). Although the authors recorded the documented medical reasons for delivery, other factors such as vaginal examinations at admission, maternal anxiety, or social concerns were not captured in the retrospective data. Due to the limitations of the present study, it is necessary to conduct studies with a prospective design or meta‐analysis studies to aggregate the results of existing studies to provide more conclusive evidence regarding the predicting factors of PPROM latency.

## 5. Conclusion

Obstetricians can expect longer latency period when PPROM cases are admitted at lower gestational age, having normal glucose tolerance status, and cervical dilation of <2 cm versus ≥ 2 dilatation at admission. Identifying predictors of latency duration can help guide obstetricians to plan for administering specific interventions (e.g., hospitalization, timing of antenatal steroids, intensive monitoring, and magnesium for neuroprotection).

**Nomenclature**
AFI: amniotic fluid index
ANOVA: analysis of variance
BMI: body mass index
CRP: C‐reactive protein
ESR: erythrocyte sedimentation rate
FHR: fetal heart rate
MMLR: multivariable multinomial logistic regression model
PPROM: preterm premature rupture of the membranes
PTL: preterm labor

## Author Contributions

Shokoh Abotorabi and Maryam Rafiei equally contributed to the conception and design of this research. Solmaz Chamanara and Zainab Alimoradi contributed to the design of this research. Maryam Rafiei and Solmaz Chamanara contributed to the acquisition. Zainab Alimoradi contributed to the analysis and interpretation of the data and drafted the preliminary version of manuscript. Mark D. Griffiths provided contributions to the literature review and discussion and prepared the final version of the manuscript, revised the final version, and copyedited the manuscript.

## Funding

No funding was received for this manuscript.

## Disclosure

All authors critically revised the manuscript, agreed to be fully accountable for ensuring the integrity and accuracy of the work, and read and approved the final manuscript to be published. All authors met the criteria for authorship and that all entitled to authorship were listed as authors.

## Ethics Statement

All the research was performed in accordance with the Declaration of Helsinki and was approved by the Institutional Review Board and Ethics Committee of Qazvin University of Medical Sciences, Qazvin, Iran (Decree Code: IR.QUMS.REC.1399.076). Due to the retrospective nature of the study, the need to obtain informed consent was waived by the Ethics Committee in Biological Research of Qazvin University of Medical Sciences.

## Consent

The authors have nothing to report.

## Conflicts of Interest

The authors declare no conflicts of interest.

## Data Availability

The data that support the findings of this study are available from the corresponding authors upon reasonable request.
